# Electroacupuncture improves endometrial receptivity and pregnancy outcomes in controlled ovarian hyperstimulation rats by restoring *Per2* circadian rhythm

**DOI:** 10.3389/fphys.2026.1812562

**Published:** 2026-10-14

**Authors:** Qian Zhu, Qian Li, Yan Zan, Tiantian Ma, Yi Fang, Yuping Sa, Tiancheng Xu, Liangjun Xia, Youbing Xia

**Affiliations:** 1 Department of Traditional Chinese Medicine, Qinghai University Medical College, Qinghai University, Xining, China; 2 Key Laboratory of Acupuncture and Medicine Research of Ministry of Education, Nanjing University of Chinese Medicine, Nanjing, China; 3 State Key Laboratory of Reproductive Medicine and Offspring Health, Nanjing Medical University, Nanjing, China

**Keywords:** circadian rhythm, controlled ovarian hyperstimulation, electroacupuncture, endometrial receptivity, implantation window

## Abstract

**Objective:**

Controlled ovarian hyperstimulation (COH) is a critical step in *in vitro* fertilization-embryo transfer (IVF-ET). However, the decline in endometrial receptivity caused by COH severely restricts the success rate of implantation. Previous studies mostly focused on static molecular events, lacking a systematic exploration from the perspective of dynamic rhythms. This study aims to explore whether electroacupuncture (EA) can improve endometrial receptivity and pregnancy outcomes by repairing the disordered rhythm of the core clock gene *Per2* in the endometrium caused by COH from the perspective of chronobiology.

**Methods:**

A COH model was established using 126 Sprague - Dawley (SD) rats. Following EA intervention, dynamic sampling was conducted during the implantation window to assess clock gene rhythmicity, the expression of receptivity - related factors, and uterine morphological changes. *Per2^−/−^* mice were used to preliminarily investigate whether *Per2* participates in mediating the effects of EA.

**Results:**

COH induced an abnormal increase in serum progesterone, leading to desynchronization of the endometrial clock gene network. Among them, the peak delay of the core clock gene *Per2* was highly coupled with the abnormal peak of progesterone in time, and presented an anti - phase oscillatory pattern with the expression of the angiogenesis factor *Vegf*. During the implantation window, the expression of *Vegf* and *integrin αvβ3* was down - regulated, the development of pinopodes degenerated, and the pregnancy rate was significantly reduced. EA could correct the abnormal progesterone level, repair the rhythm parameters of *Per2*, up - regulate the expression of *Vegf* and *integrin αvβ3*, improve the development of pinopodes, and significantly increase the pregnancy rate. In *Per2^−/−^* mice, the up - regulating effect of EA on *Vegf* and *Integrin αvβ3* was significantly weakened, preliminarily suggesting that *Per2* may participate in mediating the effects of EA on endometrial receptivity.

**Conclusion:**

This study provided preliminary evidence for a novel mechanism by which COH impairs endometrial receptivity from the perspective of dynamic circadian rhythm, providing experimental evidence for EA as an adjuvant intervention strategy based on circadian clock regulation in IVF-ET cycles.

## 1 Introduction

In the practice of *in vitro* fertilization - embryo transfer (IVF - ET), although controlled ovarian hyperstimulation (COH) can increase the number of retrieved oocytes, it often leads to a decrease in the implantation rate of fresh embryo transfer, especially in patients with high ovarian response (Check et al., 1999; Drakopoulos et al., 2023; Harvey et al., 2025). Clinical studies have shown that frozen embryo transfer can significantly improve the pregnancy outcome, indicating that the endometrial functional damage caused by COH is a key factor in implantation failure (Viganò et al., 2020; Wang et al., 2025).

Successful embryo implantation relies on the establishment of an accurate receptive state in the endometrium during the brief “window of implantation”, a process governed by a highly coordinated molecular regulatory network (Hernández-Vargas et al., 2020; Sun and Yeh, 2022). Core circadian clock genes (such as *Bmal1, Clock, Per1/2*, etc.) play important regulatory roles in the uterus and are involved in regulating cell proliferation, decidualization, and the establishment of a receptive microenvironment (Zhai et al., 2021; Zhou et al., 2025). Animal experiments further indicate that uterine circadian rhythm disorders caused by genetic or environmental factors can significantly impair embryo implantation and pregnancy maintenance, highlighting the importance of local rhythm homeostasis for reproductive success (Miller et al., 2004; Das et al., 2022). Notably, estrogen (E2) and progesterone (Pg) are known regulators of clock gene expression, capable of resetting or inducing clock gene expression (Nakamura et al., 2008; Hirata et al., 2009). Given the supraphysiological hormone levels produced by COH treatment, the disruption of the uterine circadian rhythm represents a plausible but insufficiently explored potential mechanism by which COH impairs endometrial receptivity. Previous studies on the impairment of endometrial function by COH have mostly focused on static molecular events such as signal pathway activation or changes in receptor expression (Horcajadas et al., 2005; Montoya-Botero and Polyzos, 2019), while the dynamic changes in the endometrial clock under COH conditions have not been systematically described.

Electroacupuncture (EA), a traditional Chinese medical therapy, has shown potential in improving the pregnancy outcomes of IVF-ET in recent years. Systematic reviews and meta-analyses have indicated that EA can significantly increase the clinical pregnancy rate and live birth rate (Fu et al., 2025; Peng et al., 2025). Moreover, EA has been proven to restore the circadian rhythms of multiple physiological systems (Lu et al., 2021; Wu et al., 2025), suggesting that its reproductive benefits may partly stem from the correction of the disrupted uterine rhythmic oscillations. However, there is currently a lack of direct experimental evidence to determine whether EA can improve COH-induced receptivity impairment by targeting the uterine biological clock. Based on the above background, this study hypothesizes that EA may improve endometrial receptivity and thus enhance pregnancy outcomes by restoring the rhythmic expression of uterine clock genes in COH rats. To verify this hypothesis, this study established a COH model, systematically observed the changes in pregnancy outcomes, the expression of uterine clock genes, and receptivity-related factors after EA intervention, and used *Per2^−/−^* mice to verify the dependence of the EA effect on clock genes, aiming to provide a theoretical basis for the clinical application of EA in IVF-ET.

## 2 Materials and methods

### 2.1 Animals

The experiment utilized 126 SPF-grade female Sprague Dawley (SD) rats, aged 10 weeks, weighing (220 ± 20) g, and 40 male SD rats, aged 12 weeks, weighing (400 ± 20) g. The rats were sourced from Shanghai Xipule-Bikai Laboratory Animal, a certified laboratory animal supplier (Certificate No. SCXK(Shanghai)2018-0006). Ten SPF-grade *Per2^−/−^* female mice and ten wild-type (WT) female mice, aged 10 weeks, with body weights of (20 ± 3) g, along with ten male mice weighing (25 ± 3) g, were purchased from Shanghai Southern Model Organism Technology, a company specializing in model organisms. All experimental animals were housed in the specific pathogen-free (SPF) facility of the Experimental Animal Center of Nanjing University of Chinese Medicine under strictly controlled conditions. Temperature was continuously monitored and maintained at 25 ± 2 °C and relative humidity at 55 ± 5%, using an automated climate control system. A 12-h light/dark cycle was applied, with lights on at 08:00 (Zeitgeber time 0, ZT0) and off at 20:00 (ZT12), and light intensity standardized to 150–200 lux at the cage level. Routine husbandry procedures were conducted at fixed times daily to minimize circadian disturbance, ensuring that observed circadian changes resulted from experimental interventions rather than environmental variation. This study received ethical approval from the Ethics Committee of Nanjing University of Chinese Medicine (202209A047).

### 2.2 Grouping and intervention

Following one week of adaptive feeding, estrous cycles were recorded via consecutive vaginal smears taken daily at 9:00 AM. Rats and wild-type female mice exhibiting two complete, regular estrous cycles were selected. All rats were randomly assigned to three groups using a random number table: the control group, model group, and EA group, with 42 animals per group. Wild-type female mice were assigned to the model and EA groups. *Per2^−/−^* female mice were randomly divided into the *Per2^−/−^* group and the *Per2^−/−^*EA group, with 5 animals per group. Group allocation was concealed in sealed opaque envelopes, which were opened only at the initiation of the experiment. Due to the nature of EA intervention, blinding of the operators performing the intervention was not feasible. Sample sizes for each group were determined based on pilot study data using a power calculation formula, with adequate statistical power confirmed. Five days prior to establishing the COH model, the EA group underwent isoflurane anesthesia at 21:00 daily. Following the Practical Manual of Animal Acupuncture, acupuncture interventions were performed at RN4 (2.5 cm posterior to the umbilicus) and SP6 (1 cm directly above the medial malleolus of the hind limb) acupoints. Disposable stainless steel acupuncture needles (0.18 mm × 13 mm) were used, with perpendicular insertion to a depth of 2–4 mm at RN4 and oblique insertion to approximately 5 mm at SP6. EA stimulated the RN4 and the unilateral SP6 point using a Hanz EA apparatus, alternating sides daily. Treatment lasted 15 minutes daily, with parameters set at 2/15 Hz, and the current intensity adjusted to a level where mild limb tremors were tolerable. EA continued throughout the modeling period and was discontinued on the night the modeling concluded, at which point animals were co-housed. To control for non-specific procedural effects, the control and model groups underwent identical isoflurane anesthesia and physical restraint procedures at 21:00 daily for the same duration as the EA group, without any acupuncture or electrical stimulation.

### 2.3 Establishment of the COH model and tissue sampling

After 5 days of pretreatment, rats in the model and EA groups received intraperitoneal injections of 20 U Pregnant Mare Serum Gonadotropin (PMSG) at 16:00 during the estrus interval or late estrus phase. Forty-eight hours later, an equal dose of Human Chorionic Gonadotropin (hCG) was administered. On the evening of hCG injection, they were co-housed with either normal male rats or vasectomized male rats. The following morning, successful modeling was determined by the presence of numerous sperm in vaginal smears when paired with normal males. Pairing with vasectomized males, resulting in vaginal plugs or consecutive 4-day smears showing the interestrus or late estrus phase, was also considered successful modeling. Female mice received an intraperitoneal injection of 5 U PMSG at 16:00 on the modeling day, followed by an equal dose of hCG 48 hours later. On the night of hCG injection, they were cohabited with a castrated male. Modeling success was determined by observing a vaginal plug the following morning. The control group received an intraperitoneal injection of an equal volume of saline during the same estrus cycle and was cohabited with males during estrus.

Rats that successfully mated with intact male rats were housed individually and weighed daily for 10 consecutive days. If body weight increased by 25 g or more, and if embryonic vesicles were detected by ultrasound on day 15, the longitudinal and transverse diameters of the vesicles were recorded. The rats were then kept in individual cages until delivery, and the number of pups born was recorded. For rats successfully paired with neutered males, the morning of estrus onset was defined as gestation day 0.5, and tissue and serum samples were collected every 4 hours starting at 00:00 on day 5.5 according to corresponding circadian phases: ZT16, ZT20, ZT0, ZT4, ZT8 and ZT12. Six rats per group were sampled at each time point, totaling six sampling sessions. Mice were sampled at ZT0 on day 4.5 post-estrus. During sampling, isoflurane inhalation anesthesia was administered (induction: 3–4%; maintenance: 1.5–2%), and laparotomy was performed only after confirming the absence of response to noxious stimuli. Blood was collected from the abdominal aorta. Following cervical dislocation, ovaries and uteri were dissected. Dissected tissues were processed as follows: one portion fixed in paraformaldehyde, another in glutaraldehyde solution, and the remainder stored at -80 °C. Collected blood samples were centrifuged and stored at -80 °C for subsequent use. Subsequent molecular analyses, including HE staining, IHC quantification, and Western blot, were performed independently by investigators blinded to group allocation.

### 2.4 Ultrasound examination

A large number of sperm in the vaginal smear on the morning of the second day after mating was defined as gestation day 0.5. On day 15, female rats underwent ultrasound examination, which was performed by a technician blinded to group allocation. During the procedure, the temperature and heart rate were maintained stable. Under isoflurane anesthesia, the rats were positioned in the supine position with abdominal hair shaved. The tip of the probe was coated with ultrasound gel. The scan proceeded from right to left with the probe positioned vertically against the abdominal wall in the sagittal plane, starting from the right inguinal region. Upon detecting pregnancy signs, the probe was advanced cranially along the uterine horns. When the maximum surface area of the embryonic vesicle appeared on screen, its longitudinal and transverse diameters were measured, and the average was calculated (Ypsilantis et al., 2009; Stasinopoulou et al., 2014).

### 2.5 Serum biochemical marker testing

Serum E2 and Pg levels were measured using enzyme-linked immunosorbent assay (ELISA). All kits were used according to the manufacturer’s instructions: E2 ELISA Kit (E-OSEL-R0001, Elabscience Biotech Co., Ltd., China), and Pg ELISA Kit (E-OSEL-R0007, Elabscience Biotech Co., Ltd., China).

### 2.6 Hematoxylin-eosin staining

Uterine tissue fixed in 4% paraformaldehyde was removed, trimmed, and embedded in paraffin. Paraffin blocks were sectioned into 6 μm slices, mounted onto slides, dried, and subjected to the HE staining procedure. Uterine and ovarian sections were observed using an optical microscope (Olympus, Japan).

### 2.7 Scanning electron microscopy

Fresh endometrial tissue (1 mm³) was placed at room temperature in electron microscopy fixative for 48 hours, gold-coated, and observed under a scanning electron microscope (Hitachi, Japan) to examine pinopodes structures.

### 2.8 Immunohistochemical staining

Paraffin-embedded uterine sections (6 μm) were dewaxed and rehydrated, followed by antigen retrieval in citrate buffer at elevated temperature and pressure. After cooling, tissues were blocked at room temperature with 5% goat serum to inactivate endogenous enzymes. After 30 minutes, the serum was decanted, and the tissue sections were incubated overnight at 4 °C with primary antibodies against vascular endothelial growth factor (*Vegf*, 1:200, Santa Cruz Biotechnology) and *integrin αvβ3* (1:200, Abcam). The next day, after washing with PBS, the sections were incubated with the secondary antibody at room temperature for 30 minutes. Following another PBS wash, sections were stained with 3,3’-diaminobenzidine (DAB) chromogenic buffer and counterstained with hematoxylin. Quantification of positive staining area was performed by an investigator blinded to group allocation using ImageJ software, with four randomly selected fields analyzed per section.

### 2.9 Reverse transcription quantitative real-time polymerase chain reaction

Uterine tissue was obtained at designated time points. The endometrium was scraped from the uterine cavity after dissection. Total RNA was extracted using TRIzol reagent. Following RNA concentration measurement, cDNA was synthesized using reverse transcription reagents (Yesen, China). Subsequent qRT-PCR was performed using the SYBR Green qPCR Kit (Yesen, China) on an Applied Biosystems (USA) system with a 10 μl reaction volume. Data analysis was conducted using the 2^−ΔΔCT^ method. GAPDH was used as the reference gene. Primer sequences are listed in **Supplementary Table 1**. The amplitude and phase of gene expression were determined using the CircaCompare algorithm on the *RhythmInsight* website (https://RhythmInsight.com), with rhythmicity determined at *P* < 0.05. Results are presented in **Table 1**.

**Table 1:** Cosinor analysis of circadian rhythmicity for serum hormones, endometrial clock genes, and *Vegf* during the implantation window across groups.

	Control			Model			EA		
Gene	Rhythmic(*P*)	Phase(h)	Amplitude	Rhythmic(*P*)	Phase(h)	Amplitude	Rhythmic(*P*)	Phase(h)	Amplitude
E2	0.0006	23.19	1.85	0.0018	22.31	1.61	0.1043	23.78	0.89
Pg	0.0242	13.46	1.83	0.0090	22.59	6.93	0.0806	21.58	2.95
*Bmal1*	0.0140	2.67	0.32	0.0002	13.15	0.61	0.2281	2.58	0.11
Clock	0.0267	12.08	0.53	0.1731	0.09	0.31	0.0002	11.88	0.87
*Per2*	0.0374	1.90	0.40	0.0108	23.51	0.73	0.0073	3.82	0.58
*Per1*	0.0146	4.35	0.30	0.0847	2.55	0.30	0.0122	6.12	0.32
*Vegf*	0.0223	5.41	0.86	0.0001	14.96	0.95	0.7700	3.73	0.09

*P* < 0.05 is considered a rhythmic expression.

### 2.10 Western blot

The scraped endometrial tissue was added to the lysis buffer (Beyotime) for sufficient lysis. After centrifugation, the supernatant was collected for protein concentration determination. After adjusting the concentration, the sample loading buffer was added for sufficient denaturation. The proteins were separated by SDS–PAGE and transferred to a PVDF membrane. After the membrane was blocked with a 5% bovine serum albumin solution for 1 hour, a specific primary antibody was added and incubated overnight at 4 °C. Antibodies used included: *Per2* (No. abs151542, 1:500, Absin, China), *BMAL1* (No. sc-365645, 1:500, Santa Cruz, USA), *Vegf* (No. sc-57496, 1:500 Santa Cruz, USA), and Integrin αv (No. YT2365,1:1000 Immunoway, China). After washing, the membrane was incubated in the corresponding secondary antibody solution at room temperature for 1 h. After the incubation, the membrane was washed again. Finally, chemiluminescent substrates were used for development, and the signals were detected by an imaging system. GAPDH was used as the internal reference protein, and target protein expression levels were normalized to GAPDH for analysis.

### 2.11 Statistical analysis

Data analysis was performed using GraphPad Prism 8.0 (GraphPad Inc., USA) software. Measurement data were expressed as mean ± standard deviation. Independent sample t - test was used for comparison between two groups; one - way analysis of variance (ANOVA) was used for comparison among multiple groups. The LSD method was used when the variances were homogeneous, and Dunnett’s T3 method was used when the variances were heterogeneous. Two - way repeated - measures ANOVA was used for comparison among multiple groups at multiple time points, and Bonferroni correction was used for post - hoc multiple comparisons. Two - way ANOVA was used for cross - grouping design, and Bonferroni correction was used for post - hoc multiple comparisons. Fisher’s exact test was used for comparison of rates. Spearman nonparametric test was used for the correlation analysis of variable data that did not conform to the normal distribution; one - way ANOVA or Kruskal - Wallis H test was used for count data according to the results of normality test. Differences were considered statistically significant at *P* < 0.05.

## 3 Results

### 3.1 Establishment of the COH model and effects of EA on pregnancy rates and offspring

Following the experimental protocol (**Figure 1A**), the ampulla of the fallopian tube became swollen 12 hours after superovulation, and the total number of retrieved oocytes from both sides exceeded 20 (**Figure 1B**). Compared with the control group, the number of corpora lutea in the ovary increased and the number of remaining follicles decreased in the model group (*P<* 0.05; **Figures 1C–E**), confirming the successful establishment of the COH model. Subsequently, the effect of EA on pregnancy outcomes was evaluated (**Table 2**). The pregnancy rate in the model group was significantly lower than that in the control group, while EA treatment could effectively increase the pregnancy rate (*P* < 0.05, **Figure 1F**). In addition, ultrasound examination on the 15th day of pregnancy (**Figures 1G, H**) showed no obvious morphological abnormalities in the embryos of the three groups, and there was no significant difference in the average diameter of the embryonic vesicles, preliminarily suggesting that assisted reproduction and EA treatment had no adverse effects on the mid - term development of embryos.

**Figure 1:**
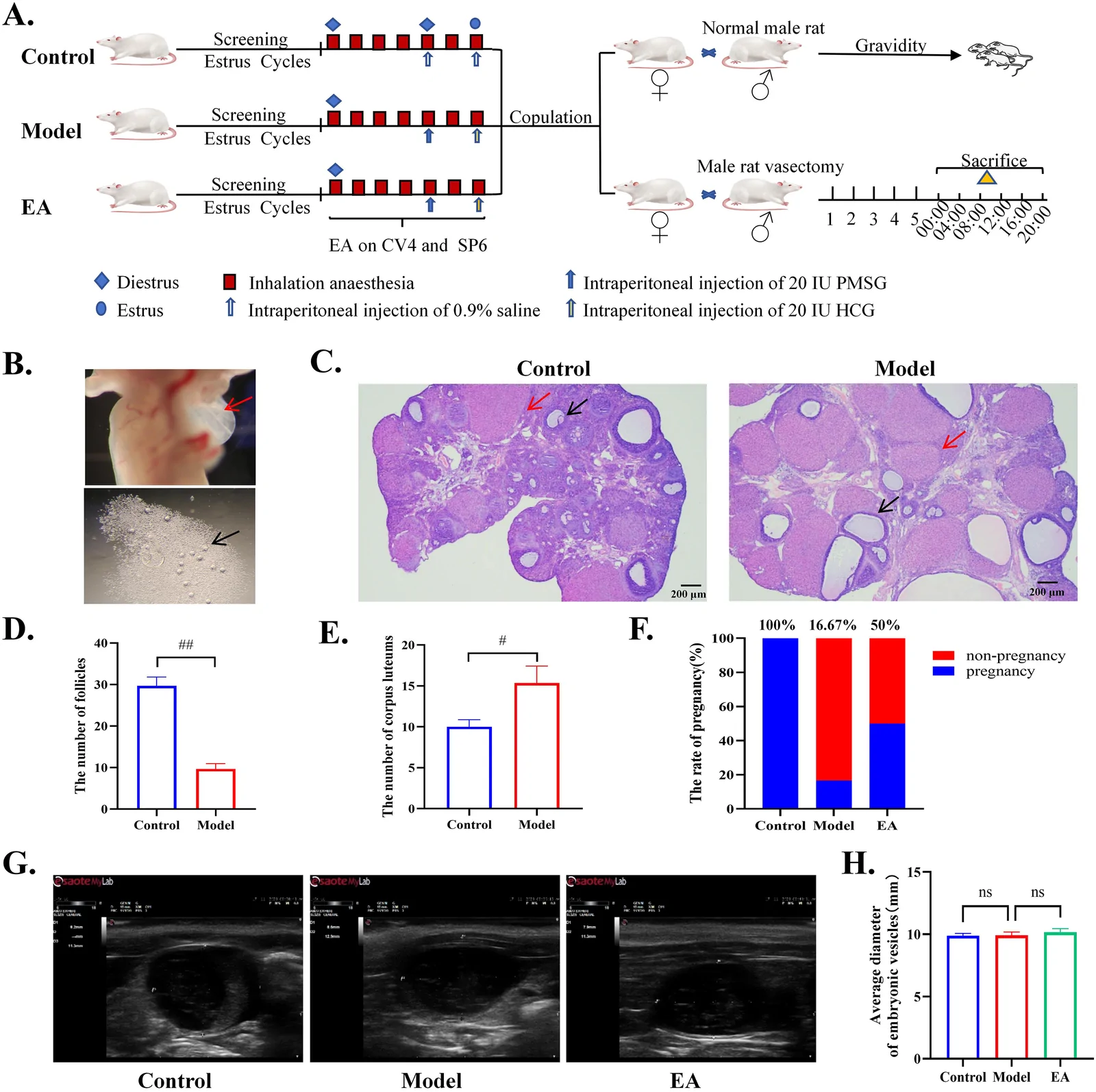
Establishment of the COH Rat model and evaluation of EA effects on pregnancy rates and offspring development. **(A)** Experimental workflow diagram; **(B)** Enlarged ampulla of the fallopian tube and follicles retrieved from one side of the ampulla on the second day after successful cohabitation. Red arrows indicate the enlarged ampulla; black arrows indicate follicles; **(C)** HE-stained ovarian sections on day 5 post-successful mating, original magnification ×40.Red arrows indicate corpora lutea; black arrows indicate follicles; **(D)** Corpus luteum count in ovaries on day 5 post-mating (n=6); **(E)** Follicle count in ovaries on day 5 post-mating (n=6); **(F)** Pregnancy rate percentage across groups (n=6); **(G)** Ultrasound morphology of embryos at mid-gestation (gestation day 15); **(H)** Mean diameter of embryonic vesicles ( $\bar{x}\pm \text{s}$, n=6). COH, Controlled Ovarian Hyperstimulation. ^#^*P* < 0.05 vs. Control. ^##^*P* < 0.01 vs. Control.

**Table 2:** Comparison of pregnancy outcomes among the groups of rats.

Group	Mated (n)	Pregnant (n)	Mean litter size	Total pups (n)	Pregnancy rate (%)
Control	6	6	11.00 ± 1.41	66	100.00
Model	6	1	2.83*	17	16.67
EA	6	3	6.17 ± 6.80	37	50.00

*Only one rat in the model group became pregnant, so the standard deviation could not be calculated.

### 3.2 Effects of EA on uterine morphology during the implantation window in rats

To exclude the interference of hormones and growth factors secreted by the embryos themselves on the maternal uterine environment, subsequent experiments were all performed using pseudopregnant rats mated with vasectomized male rats for analysis. Their hormone levels and physiological responses were similar to those in the early pregnancy stage. HE results showed that during the implantation window period, the uterine cavity area and endometrial thickness in the control group increased in a time - dependent manner. The model group showed excessive uterine dilation at ZT16 (*P<* 0.01), followed by rapid atrophy; while the EA group could still maintain a relatively dilated uterine morphology after ZT20 (*P<* 0.05; **Figures 2A–C**). This “premature dilation - rapid atrophy” pattern in the model group suggested impaired temporal regulation of the endometrium, and EA could partially correct this abnormality. To elucidate its underlying mechanism, we further detected the circulating hormone levels and the expression rhythm of local clock genes in the uterus during the implantation window period.

**Figure 2:**
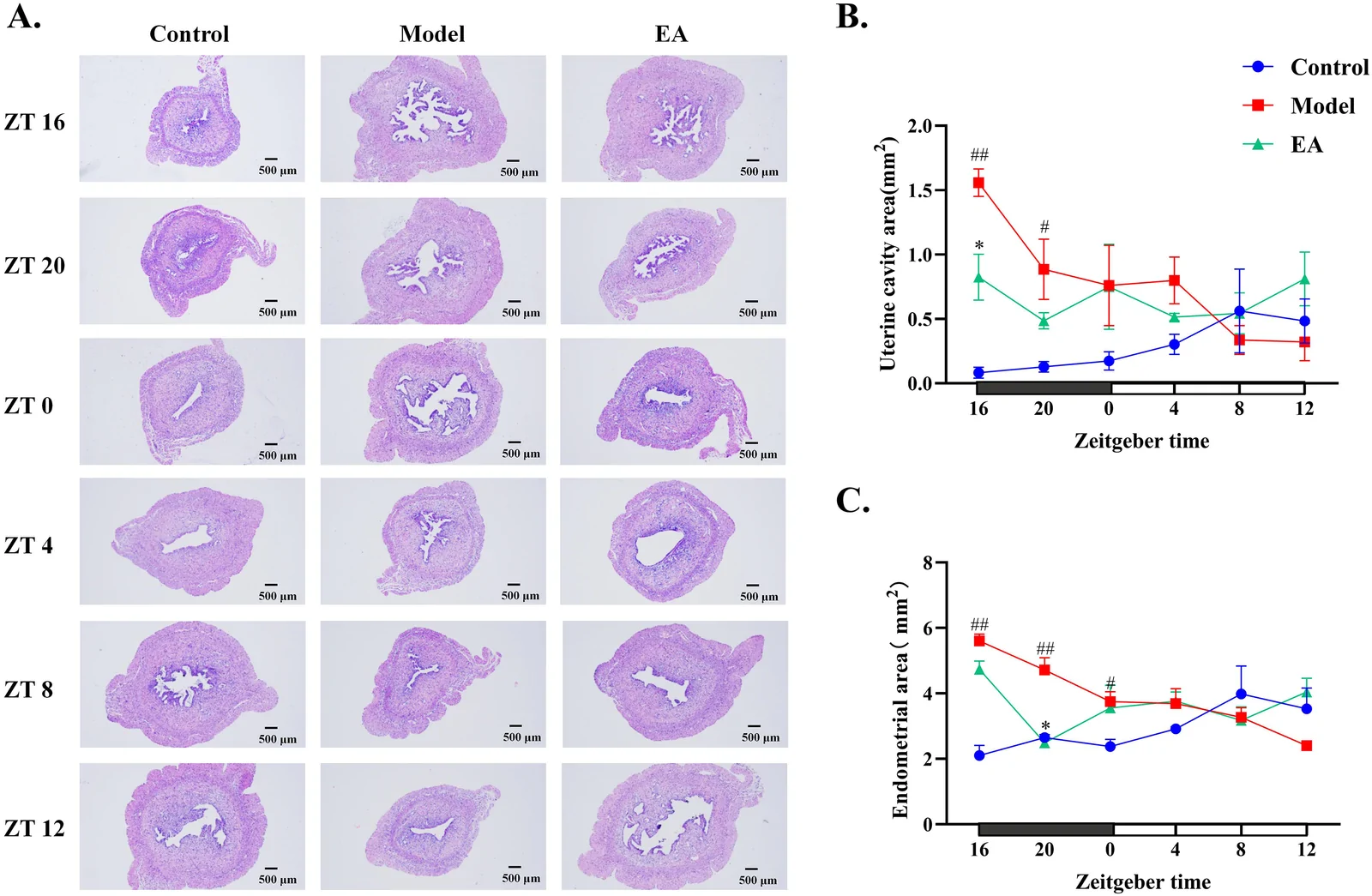
Morphological changes in rat uterine tissue at different time points during the implantation window following EA. **(A)** HE-stained images of rat uterine tissue at various time points. Original magnification ×40; **(B)** Changes in uterine cavity area during the implantation window among groups. Black bars on the horizontal axis indicate the dark phase, and white bars indicate the light phase ( $\bar{x}\pm \text{s}$, n=5); **(C)** Changes in endometrial area during the implantation window across groups ( $\bar{x}\pm \text{s}$, n=5). ^#^*P* < 0.05 vs. Control. ^##^*P* < 0.01 vs. Control. *P < 0.05 vs. Model.

### 3.3 Effects of EA on serum E2 and Pg levels and core clock gene expression in rats during the implantation window period

Serum hormone analysis showed that both E2 and Pg exhibited circadian rhythms in the control group. No significant differences in E2 phase or amplitude were observed among the three groups, suggesting that COH had limited effect on the circadian rhythm of serum E2 during the implantation window, and EA did not exert significant regulatory effects on E2 rhythmicity. Compared with the control group, the model group had an increased amplitude and a delayed phase of Pg, which temporally corresponded to the morphological changes of premature endometrial dilation. EA could normalize the amplitude of Pg without altering its phase (**Figures 3A, B**).

**Figure 3:**
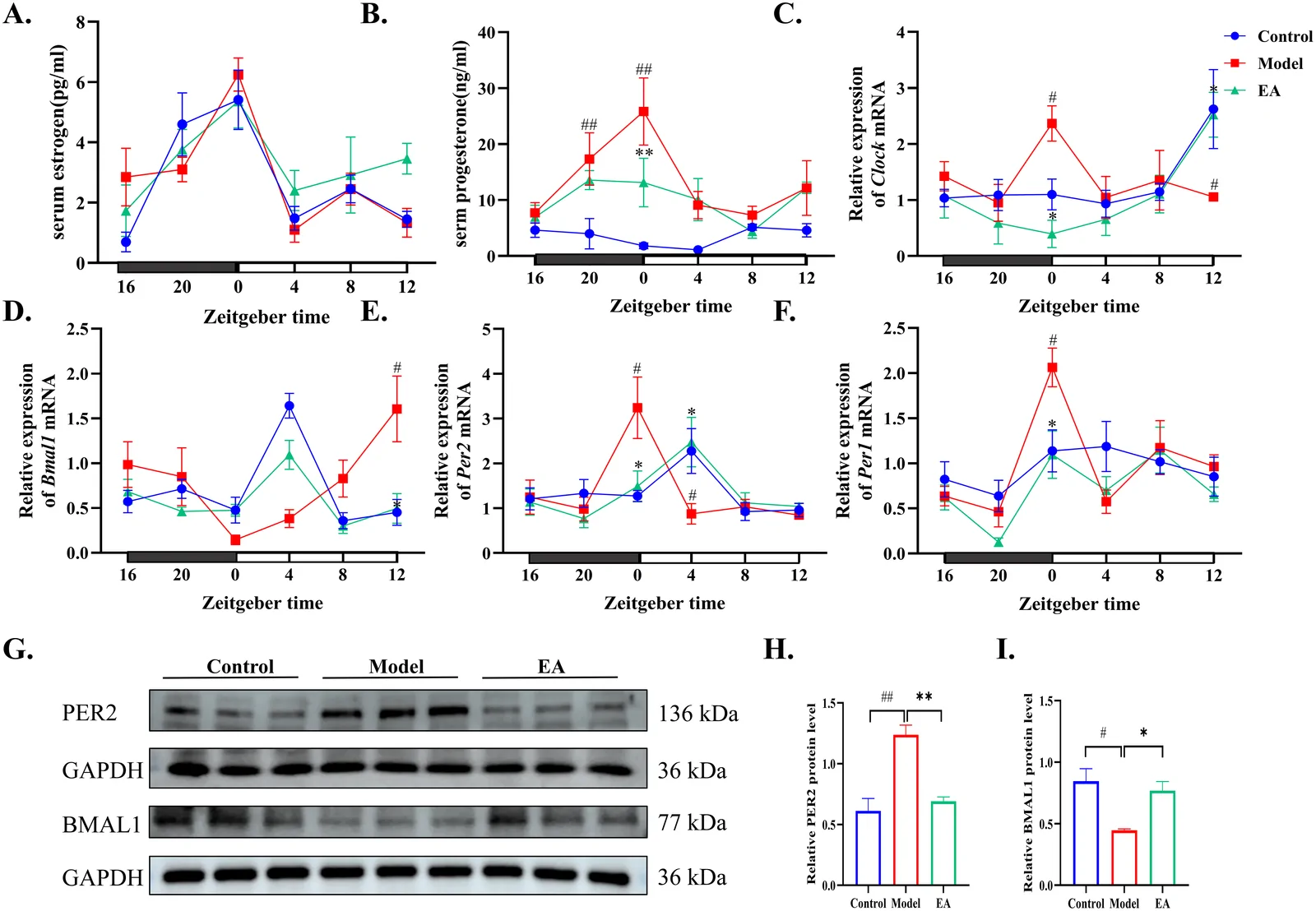
Effects of EA on serum estrogen and progesterone levels and core circadian clock gene expression. **(A)** Serum estrogen expression. ( $\bar{x}\pm \text{s}$, n=5); **(B)** Serum progesterone expression. ( $\bar{x}\pm \text{s}$, n=5); **(C–F)** Dynamic mRNA expression of uterine core circadian clock genes *Clock, Bmal1*, *Per2*, and *Per1*. ( $\bar{x}\pm \text{s}$, n=5); **(G–I)** Protein expression of uterine core clock genes *Per2* and *Bmal1*. ( $\bar{x}\pm \text{s}$, n=3). Clock, Circadian Locomotor Output Cycles Kaput; *Bmal1*, Brain and Muscle ARNT-Like1; *PER2*, Period Circadian Regulator2; *Per1*, Period Circadian Regulator1. ^#^*P* < 0.05 vs. Control. ^##^*P* < 0.01 vs. Control. *P < 0.05 vs. Model. **P < 0.01 vs. Model.

The results of the rhythm analysis of core clock genes in the endometrium showed that the circadian rhythm was disrupted in the model group (**Table 1**), specifically manifested as advanced phases and weakened amplitudes of *Clock* and *Per1*, as well as delayed phases and increased amplitudes of *Bmal1* and *Per2*. Following EA intervention, the phase shift and amplitude abnormalities of *Per2* were significantly corrected (*P<* 0.05); rhythmic parameters of *Clock* and *Per1* were also partially restored (*P<* 0.05); however, the rhythmicity of *Bmal1* did not reach statistical significance (*P* > 0.05) (**Figures 3C–F**). Notably, the peak of *Per2* in the model group was temporally coupled with the peak of Pg, and EA intervention could synchronously correct the abnormalities in the phase and amplitude of *Per2*. At ZT0, a time point showing relatively prominent intergroup differences, Western blot analysis further confirmed the protein expression changes of *Per2* and *Bmal1* (**Figure 3G**). COH exposure increased the protein level of *Per2* (*P* < 0.01; **Figure 3H**) and decreased the expression of *BMAL1* (*P* < 0.05; **Figure 3I**), while EA intervention could significantly improve these conditions (*P* < 0.05).

### 3.4 Effects of EA on the expression of endometrial receptivity in rats during the implantation window period

Given that prior studies have suggested a potential negative regulatory relationship between *Per2* and *Vegf*, we further examined the dynamic expression of *Vegf* to explore whether such an association exists in the endometrium. The results showed that *Vegf* expression exhibited circadian rhythmicity in both the control and model groups. In the model group, *Per2* and *Vegf* showed an obvious reverse - phase circadian rhythm pattern. Compared with the control group, the phase of the model group was significantly shifted. Following EA intervention, rhythmicity was lost; however, *Vegf* expression levels were continuously upregulated and maintained at a relatively high and stable state (**Figure 4A**). WB at ZT0 confirmed that the levels of *Vegf* and Integrin αv proteins were significantly decreased in the model group, and EA could effectively reverse this (*P* < 0.05; **Figures 4B, C**). Immunohistochemistry showed that the positive area of *integrin αvβ3* in the luminal and glandular epithelia was reduced in the model group, and EA significantly restored its expression (*P* < 0.01; **Figures 4D, E**). Scanning electron microscopy showed that the control group had abundant pinopodes, while in the model group, the pinopodes were collapsed and atrophied, and EA partially restored them (**Figure 4F**).

**Figure 4:**
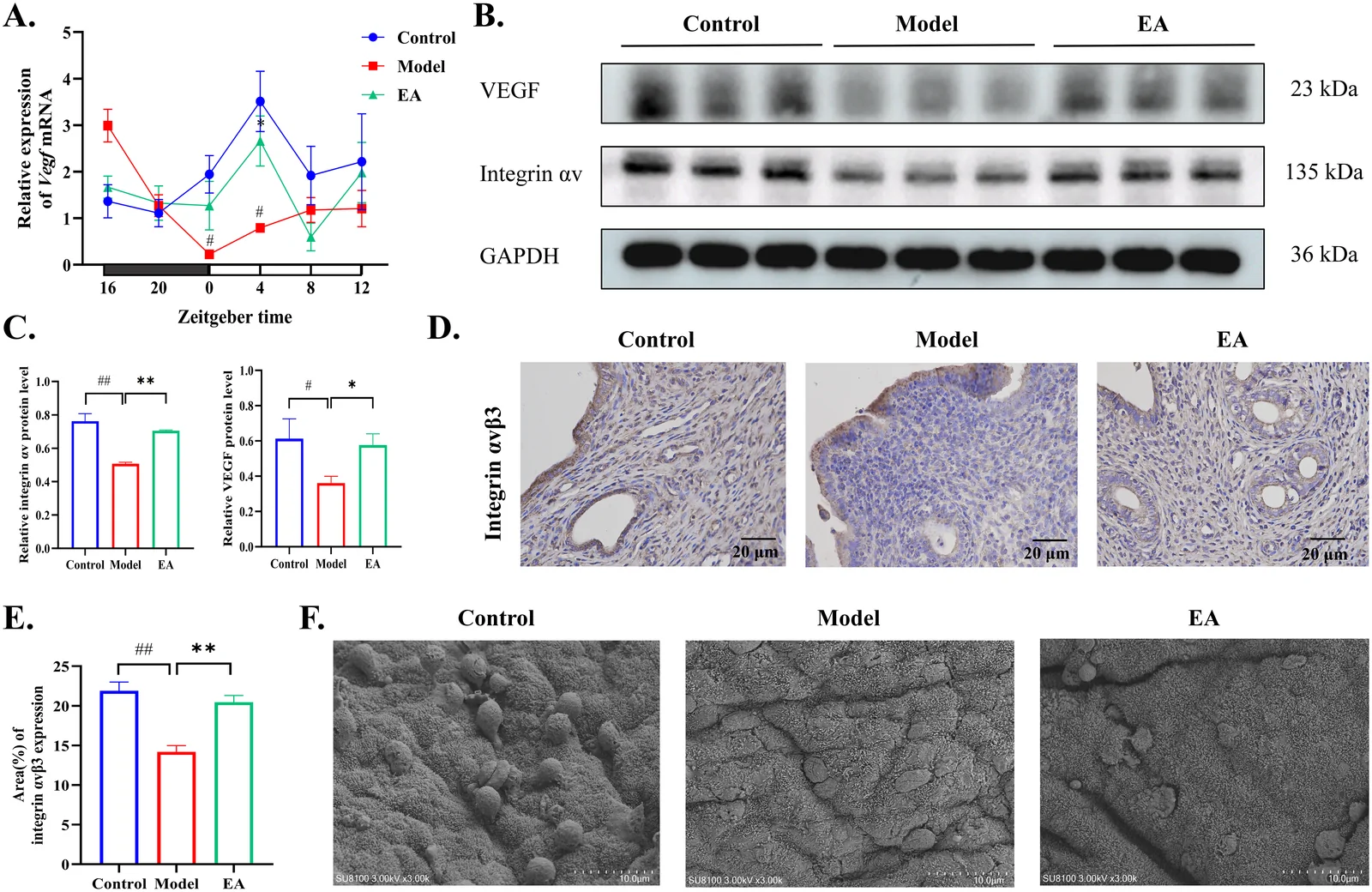
Effects of EA on endometrial receptivity expression in rats. **(A)** Dynamic expression of *Vegf* mRNA in the endometrium. ( $\bar{x}\pm \text{s}$, n=5); **(B)** Western blot images of uterine *integrin αv* and *Vegf* expression. (n=3); **(C)** Quantitative analysis of Integrin αv and *VEGF* protein expression. ( $\bar{x}\pm \text{s}$, n=3); **(D, E)** IHC staining images and semi-quantitative analysis of *integrin αvβ3* complex. Original magnification ×400; **(F)** Expression of pinopodes in rats from each group under scanning electron microscopy. Original magnification ×3000. *Vegf*, Vascular Endothelial Growth Factor; integrin αv, integrin alpha V; *integrin αvβ3*, integrin alpha V beta 3. ^#^*P* < 0.05 vs. Control. ^##^*P* < 0.01 vs. Control. *P < 0.05 vs. Model. **P < 0.01 vs. Model.

### 3.5 Effects of EA on the expression of endometrial receptivity in *Per2^−/−^* mice

Given that *Per2* rhythmic expression in *Per2^−/−^* mice has been lost, this part of the experiment aims to preliminarily investigate whether *Per2* participates in mediating the effects of EA rather than the rhythmic dynamic changes. Therefore, a single time point, ZT0, with relatively prominent intergroup differences, was selected for comparison. HE staining showed no significant difference in endometrial thickness among groups (**Figures 5A, B**). Serum Pg increased in the wild - type model group, and EA could reduce it (*P* < 0.01); the increase in Pg in *Per2^−/−^* mice was attenuated (*P* < 0.05), and there was no significant change after EA intervention (**Figure 5C**). EA significantly up - regulated the expression of *Vegf* and *integrin αvβ3* in the wild - type model group (*P* < 0.01). In *Per2^−/−^* mice, EA intervention also up - regulated their expression, but to a lesser extent (*P* < 0.05; **Figures 5D, E**).

**Figure 5:**
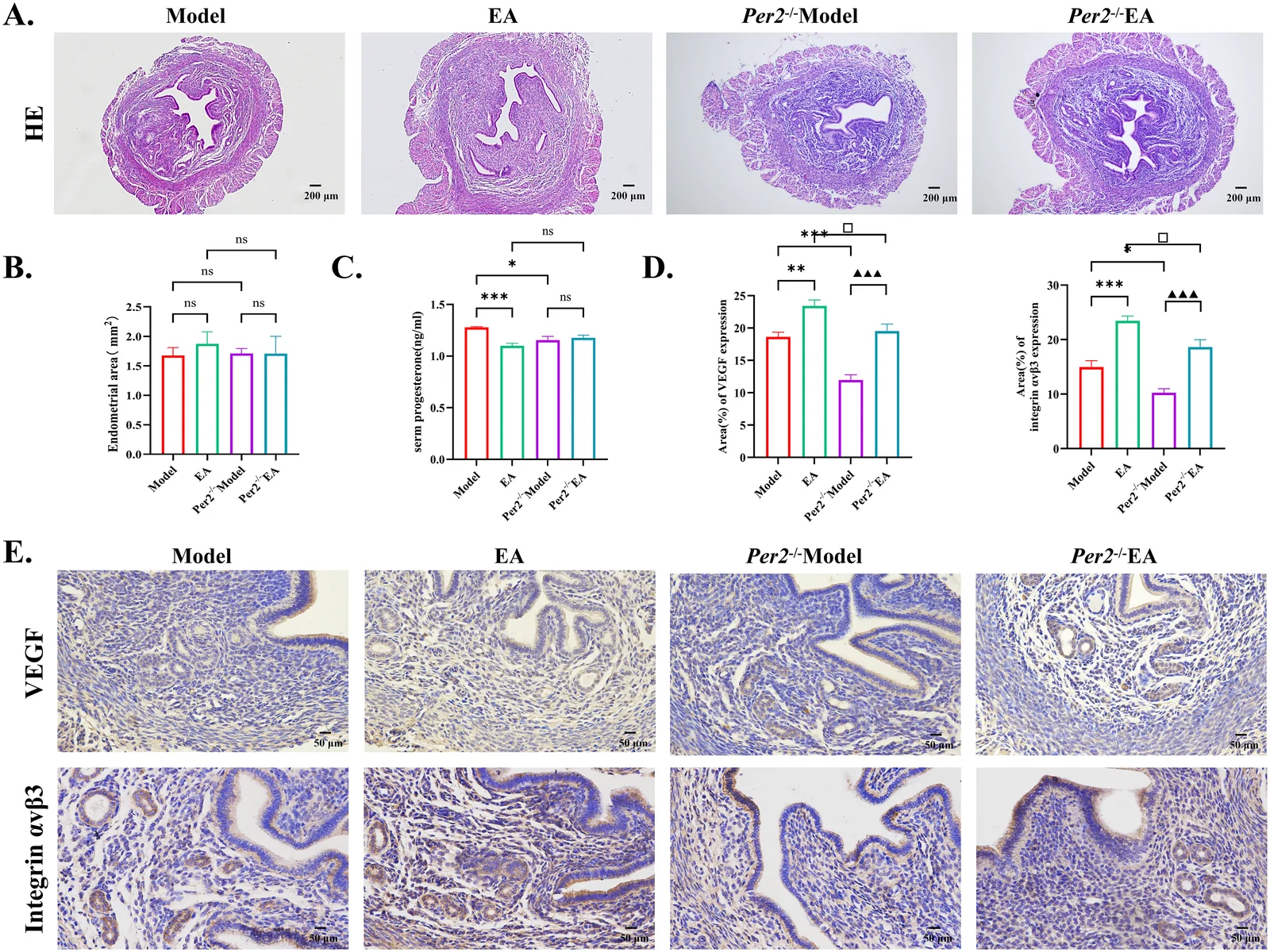
Effects of EA on endometrial receptivity expression in *Per2^-/-^* Mice. **(A)** HE staining images of uterine tissue from each group, original magnification ×100; **(B)** Endometrial area in each group; **(C)** Serum progesterone levels ( $\bar{x}\pm \text{s}$, n=5); **(D, E)** IHC staining images and semi-quantitative analysis of *VEGF* and *Integrin αvβ3*, original magnification ×400, ( $\bar{x}\pm \text{s}$, n=5). ^*^P < 0.05 vs. Model. ^**^P < 0.01 vs. Model. ^***^P < 0.001 vs. Model. ^▲▲▲^*P* < 0.001 vs. *PER2^-/-^*Model. ^Δ^*P* < 0.05 vs. EA.

## 4 Discussion

The core finding of this study is that the damage of COH to the endometrium is not limited to the expression change of a single molecule, but involves the comprehensive disorder of the temporal structure of the clock gene network. Following EA intervention, the rhythmic parameters of the core clock gene *Per2* were restored, temporal disruption was improved, and the pregnancy rate was significantly increased. The bidirectional evidence of this damage and repair together supports the inference that the endometrial biological clock plays an important role in the regulation of embryo implantation. Different from the studies on congenital clock gene defects (Zheng et al., 2001; Boden et al., 2010), this study suggests that the supraphysiological hormone exposure caused by COH may also lead to the dysfunction of the uterine clock gene network and damage implantation. The effective correction of this rhythmic disorder by EA further expands the understanding of the role of clock genes in reproductive regulation.

The initiating factor for the rhythm disorder caused by COH points to supraphysiological steroid hormone levels. Nakamura et al. found that under the condition of a constant E2 concentration, high - concentration Pg could dose - dependently delay the peak phase of uterine *Per2* and increase its amplitude (Nakamura et al., 2010). Hirata et al. also confirmed that Pg could synchronize the Per2 rhythm oscillation of endometrial stromal cells (Hirata et al., 2009). In this study, the serum Pg in the COH model group showed rhythm characteristics of a significantly increased amplitude accompanied by a phase delay, and its abnormal peak was highly coupled with the peak of endometrial *Per2*, which was consistent with the above literature reports. Collectively, it is inferred that the supraphysiological hormone fluctuations during COH may influence the synchronization of the endometrial circadian oscillator by altering the phase and intensity of zeitgeber signals; however, this inference awaits further validation through *in vitro* mechanistic studies.

EA intervention precisely targets this initiating step. After EA treatment, the excessive amplitude of serum Pg in the model group was significantly corrected, and the phase shift and abnormal amplitude of uterine *Per2* were synchronously improved. The synergistic effect between the normalization of Pg amplitude and the restoration of *Per2* rhythm supports the inference that abnormal Pg rhythm serves as the initiating factor of uterine clock disorder. Notably, EA selectively corrected the amplitude rather than the phase of Pg, suggesting that its mechanism of action may lie in inhibiting the excessive activation of luteal function caused by COH to attenuate the abnormal peak, rather than resetting the secretion timing of steroid hormones. Previous studies have confirmed that EA can inhibit the excessive luteinization reaction by regulating the function of the hypothalamus - pituitary - ovary axis (Huang et al., 2018; Zhu et al., 2019). The results of this study are consistent with those findings. The complete disappearance of the regulatory effect of EA on serum Pg in *Per2^−/−^* mice further suggests that the regulation of circulating hormones by EA may partly depend on the integrity of the peripheral clock. Considering that clock genes in the ovary are involved in regulating the rhythmic expression of steroid - synthesizing enzymes (Fahrenkrug et al., 2006; Ratajczak et al., 2009), a bidirectional feedback relationship between Pg and *Per2* may exist, whereby *Per2* is not only a downstream target of Pg signaling but may also reciprocally influence circulating Pg levels by modulating ovarian steroidogenesis; however, this remains speculative and awaits further experimental validation. This hormonal challenge triggered a notable differential response in the clock gene network: Rhythm analysis showed that *Clock* and *Per1* exhibited phase advancement accompanied by amplitude attenuation, while *BMAL1* and *Per2* showed phase delay accompanied by amplitude enhancement. In the normal molecular clock loop, the *Clock/Bmal1* heterodimer drives the transcription of *Per/Cry* genes, and their protein products feedback - inhibit the activity of *Clock/Bmal1*, forming an approximately 24 - hour oscillation cycle (Takahashi, 2017; Ono et al., 2023). The differential response of clock genes observed in this study means that the coupling relationship within this loop is disrupted, rather than a simple enhancement or weakening of the overall oscillation, indicating that the effect of COH on clock genes is not a single “rhythm - inhibition” pattern. This finding echoes the results of Nakamura et al., who observed tissue - specific and gene - specific changes in clock gene expression at different stages of the estrous cycle *in vivo* (Nakamura et al., 2010).

The abnormal hyperactivity of *Per2* deserves special attention because it is directly associated with the impairment of the receptive microenvironment during the implantation window (Muter et al., 2015; Zhang et al., 2019; Zheng et al., 2019), which is precisely the key target layer where EA exerts its therapeutic effects. Previous studies have shown that *Per2* can inhibit *Vegf* transcription by promoting the degradation of HIF-1α, and this mechanism has been confirmed in various cell models (Koyanagi et al., 2003; Jensen et al., 2012). Given that the HIF-1α/*Vegf* axis is the core regulatory pathway for endometrial vascular remodeling and the establishment of receptivity (Maybin and Critchley, 2015; Guo et al., 2021; Zehorai et al., 2024), the inverse oscillatory pattern of *Per2* and *Vegf* observed in this study suggests that this regulatory relationship may also exist in the endometrium. The rhythmic data in this study provide dynamic evidence for this regulatory relationship: in the model group, *Per2* and *Vegf* exhibited a clear anti - phase oscillation. The period when the *Per2* amplitude increased abnormally overlapped with the period when *Vegf* expression was suppressed, suggesting that the rhythmic hyperactivity of *Per2* may be associated with the attenuation of endometrial vascularization support during the implantation window; however, the directional regulatory relationship between the two requires further experimental confirmation. EA’s repair of the *Per2* rhythm effectively relieved this excessive inhibition, and the expression levels of *Vegf* and *integrin αvβ3* both returned to near - control levels. Consistent with the aforementioned molecular changes, the positive expression area of *integrin αvβ3* in the luminal and glandular epithelia of the model group was significantly reduced compared to the control group. Under scanning electron microscope, the pinopodes showed collapse and atrophy, while after EA treatment, the pinopodes re-exhibited their mature morphology. The maturity of pinopodes is considered an important morphological marker of endometrial receptivity (Melkozerova et al., 2019; Zhu et al., 2025), and its formation is functionally closely associated with the up - regulation of *Vegf* and *integrin αvβ3* expression. Although the evidence for the direct transcriptional regulation of integrins by *Per2* is insufficient, *Vegf* can promote the expression and activation of integrins through paracrine signals (Byzova et al., 2000; Lee et al., 2025); therefore, the suppression of *Vegf* accompanying *Per2* rhythmic abnormality may represent one upstream correlate of the down-regulation of *integrin αvβ3*. Morphologically, the uterus in the model group showed an abnormal temporal pattern of premature expansion and rapid atrophy during the implantation window, further supporting the inference of impaired endometrial temporal regulation. Taken together, the rhythmic disorder of *Per2* may affect multiple receptive dimensions such as vascular remodeling, epithelial adhesion, and luminal morphological maturation through *Vegf* as a hub node, constituting a cascade damage network in which clock genes serve as the upstream driver. EA achieved the overall improvement of the receptive microenvironment by repairing the upstream driving node of this network.

Experiments on *Per2^−/−^* mice provided preliminary directional support for the above inference: after *Per2* deficiency, the up - regulation of *Vegf* and *integrin αvβ3* by EA was significantly weaker than that in WT mice, suggesting that the presence of *Per2* is important for EA’s effect of improving endometrial receptivity, and supporting the possibility that *Per2* participates in mediating this process. Notably, EA still retained a partial up - regulation effect in *Per2^−/−^* mice, indicating the possible existence of non-*Per2*-dependent parallel pathways. Furthermore, *Vegf* in the EA group did not resume rhythmic oscillation but maintained a stable and high expression level. Whether sustained high *Vegf* expression during the implantation window is more conducive to endometrial vascular remodeling than rhythmic oscillation remains to be clarified by future studies. These observations suggested that the improvement of the receptive microenvironment by EA might not be achieved by mechanically restoring the rhythmic expression of each molecule, but by correcting the abnormality of upstream rhythmic parameters, enabling downstream effector molecules to maintain a functional state favorable for implantation.

However, this study still has limitations. This study did not include a sham acupuncture control group (such as non-acupoint shallow needling or manual acupuncture), and therefore cannot fully distinguish the acupoint-specific effects of EA from non-specific stimulation effects. Future studies should incorporate a sham acupuncture group to further verify the acupoint dependence of EA effects. In addition, the sample size of *Per2^−/−^* mice was relatively limited due to strain accessibility, and they constitute a systemic gene knockout model, which cannot rule out the indirect interference of *Per2* deficiency on other tissues such as the ovaries on the experimental results. The existing conclusions represent preliminary evidence for the *Per2* dependence of EA effects, rather than direct proof of local endometrial *Per2*-specific mediation, and further verification using larger sample sizes and uterus-specific conditional knockout models is needed. Meanwhile, this study revealed a dynamic inverse relationship between *Per2* and *Vegf*, but the direct regulatory mechanism between the two in endometrial cells still needs to be confirmed by *in vitro* experiments such as ChIP or luciferase reporter gene assays. In addition, the Western blotting verification was only performed at a single time point (ZT0), failing to present the complete rhythmic dynamics at the protein level. The rhythmic changes of *Per2* and *Vegf* proteins need to be confirmed by subsequent time-series proteomic studies. Future research should progressively advance in these directions.

## 5 Conclusion

This study suggested that COH disrupts the circadian rhythm of the core clock gene *Per2* in the endometrium by inducing abnormal supraphysiological progesterone rhythms. Concomitantly, the expression of *Vegf* is inhibited and shows a dynamic inverse relationship with *Per2*. Meanwhile, the expression of *integrin αvβ3* and the maturity of pinopodes decrease. EA intervention can correct the amplitude hyperactivity of progesterone, repair the rhythm parameters of *Per2*, restore the expression of downstream receptivity molecules and the morphology of the uterine luminal surface, and significantly improve pregnancy outcomes. Experiments on *Per2^−/−^* mice preliminarily suggested that *Per2* may participate in mediating the above effects of EA. This study provided a new perspective for understanding the potential mechanism by which COH impairs endometrial receptivity from the dimension of dynamic rhythm, and provided preliminary experimental evidence for EA as a non-pharmacological adjuvant intervention strategy based on circadian clock regulation in IVF-ET cycles.

## Data Availability

The original contributions presented in the study are included in the article/**Supplementary Material**. Further inquiries can be directed to the corresponding authors.
