# Supplementary material for: Electroacupuncture improves endometrial receptivity and pregnancy outcomes in controlled ovarian hyperstimulation rats by restoring Per2 circadian rhythm
Source: Front Physiol. 2026 Oct 14;17:1812562. doi: 10.3389/fphys.2026.1812562 (PMC13713282; doi:10.3389/fphys.2026.1812562)
Supplement: Supplementary file 1 [file DataSheet1.docx]

**Table 1. Primer Sequences for qRT-PCR**

| Gene | Accession number | Sequence (5′→3′) | Amplicon (bp) |
| --- | --- | --- | --- |
| VEGF | NM_001110333.2 | F: CCTGGCTTTACTGCTGTACCT | 110 |
|  |  | R: GCTGGTAGACGTCCATGAACT |  |
| CLOCK | NM_001289832.2 | F: ACGGCGAGAACTTGGCATTGAG | 117 |
|  |  | R: AACCTTTCCAGTGCTTCCTTGAGAC |  |
| BMAL1 | NM_024362.2 | F: AGGACTTCGCCTCCACCTGTTC | 137 |
|  |  | R: GCCCTCATTGTCTGGTTCACTGTC |  |
| PER1 | NM_001034125.1 | F: GCCTGGCTGATGACACTGATGC | 100 |
|  |  | R: GAACTGTGAGAACTCCGCTGAGATG |  |
| PER2 | NM_031678.2 | F: CGCCATCCACAAGAAGATCCTACAG | 133 |
|  |  | R: CCTGCTCCACGGGTTGATGAAG |  |
| GAPDH | NM_017008.4 | F: GGCACAGTCAAGGCTGAGAATG | 143 |
|  |  | R: ATGGTGGTGAAGACGCCAGTA |  |

**Full uncropped Gels and Blots image(s)**

| **PER2** |  |
| --- | --- |
| **BMAL1** |  |
| **VEGF+Integrin αv** |  |
|  |  |
|  |  |
